# Euarchontoglires Challenged by Incomplete Lineage Sorting

**DOI:** 10.3390/genes13050774

**Published:** 2022-04-27

**Authors:** Liliya Doronina, Olga Reising, Hiram Clawson, Gennady Churakov, Jürgen Schmitz

**Affiliations:** 1 Institute of Experimental Pathology, ZMBE, University of Münster, 48149 Münster, Germany; o_reis01@uni-muenster.de (O.R.); churakov@uni-muenster.de (G.C.); jueschm@uni-muenster.de (J.S.); 2 Department of Biomolecular Engineering, University of California, Santa Cruz, CA 95064, USA; hiram@soe.ucsc.edu; 3 EvoPAD-RTG, University of Münster, 48149 Münster, Germany

**Keywords:** Euarchontoglires, transposed elements (TEs), presence/absence, GPAC, 2-n-way, 4-lineage statistical test, retrophylogenomics, ancestral incomplete lineage sorting

## Abstract

Euarchontoglires, once described as Supraprimates, comprise primates, colugos, tree shrews, rodents, and lagomorphs in a clade that evolved about 90 million years ago (mya) from a shared ancestor with Laurasiatheria. The rapid speciation of groups within Euarchontoglires, and the subsequent inherent incomplete marker fixation in ancestral lineages, led to challenged attempts at phylogenetic reconstructions, particularly for the phylogenetic position of tree shrews. To resolve this conundrum, we sampled genome-wide presence/absence patterns of transposed elements (TEs) from all representatives of Euarchontoglires. This specific marker system has the advantage that phylogenetic diagnostic characters can be extracted in a nearly unbiased fashion genome-wide from reference genomes. Their insertions are virtually free of homoplasy. We simultaneously employed two computational tools, the genome presence/absence compiler (GPAC) and 2-n-way, to find a maximum of diagnostic insertions from more than 3 million TE positions. From 361 extracted diagnostic TEs, 132 provide significant support for the current resolution of Primatomorpha (Primates plus Dermoptera), 94 support the union of Euarchonta (Primates, Dermoptera, plus Scandentia), and 135 marker insertion patterns support a variety of alternative phylogenetic scenarios. Thus, whole genome-level analysis and a virtually homoplasy-free marker system offer an opportunity to finally resolve the notorious phylogenetic challenges that nature produces in rapidly diversifying groups.

## 1. Introduction

The historically uncertain phylogenetic position of rodents within mammals was finally resolved by the groundbreaking phylogenetic study of Murphy et al. [1]. Subsequent confirmation of the rodent-including clade Euarchontoglires (synonym Supraprimates [2]) and their position within mammals was implemented by diagnostic presence/absence patterns of transposed elements (TEs) [3]. Along with rodents (e.g., mice, guinea pigs), the superorder Euarchontoglires is comprised of lagomorphs (hares and rabbits), primates (e.g., humans, lemurs), dermopterans (colugos), and scandentians (tree shrews). The decades-long inconsistent phylogenetic positions of dermopterans and scandentians within Euarchontoglires (for example [1]) probably indicate extensive molecular sequence noise that is likely due to unfixed markers resulting from speciation during rapid radiation. This issue is well summarized in the colugo (*Galeopterus variegatus*) genome study, which confirmed the positions of primates and dermopterans in the monophyletic group Primatomorpha [4]. However, several studies provided conflicting evidence for the positions of especially scandentians within Euarchontoglires. Song et al. [5], Fan et al. [6], and Kumar et al. [7] showed Scandentia to be closest to Primates (unfortunately, Dermoptera were not included in any of these studies), while Nishihara et al. [8] provided TE presence/absence evidence for a close relationship between Scandentia and rodents. At the same time, mitochondrial genome sequences indicated some affinity of Scandentia to Lagomorpha [9] or Glires [10], and Murphy et al. [11] demonstrated the merging of Dermoptera and Scandentia in the group Sundatheria (see also [12]). Subsequently, Zhou et al. [13] suggested that the Euarchontoglires relationship represents a case of hard polytomy. They predicted that whole-genome analyses would not resolve the position of tree shrews within Euarchontoglires.

Taking advantage of the current availability of genomes from all representatives of Euarchontoglires orders/suborders, we decided to revisit this question by employing new computational tools to conduct the first genome-level analyses of TE phylogenetic presence/absence signals and visualize their various conflicting insertion patterns. There are many advantages of using this special marker system to resolve such complicated phylogenies, and they have been successfully employed in analyzing the evolutionary relationships of many different animal groups (e.g., Afrotheria [14]; Dermoptera [4]; Laurasiatheria [15]; Aves [16]). The ancestral genomic insertion of a TE in a common progenitor and the simultaneous passage of the fixed elements into two diverging species irreversibly marks their close relationship compared to unrelated species that exhibit an empty orthologous target site. Nevertheless, as is true of any marker system, when the period between insertion/change and speciation is too short for fixation (e.g., less than a few million years for primates [17]) due to rapid species radiation, such unfixed TEs tend to distribute randomly within species (e.g., [18]). This ancestral incomplete lineage sorting (ILS) is often what leads to the controversial signals observed in present-day species affiliations. In some cases, successive radiations during short periods might lead to multiple conflicting TE patterns that may obscure the true phylogenetic signal [19]. However, due to their virtually homoplasy-free character [20,21], TE insertion patterns enable us to visualize all irregular patterns of ILS. Furthermore, alternating four different reference species (1) human (*Homo sapiens*) for primates, (2) colugo (*G. variegatus*) for dermopterans, (3) Chinese tree shrew (*Tupaia belangeri chinensis*) for scandentians, and (4) mouse (*Mus musculus*) for rodents (see Materials and Methods) in separate runs enables us to detect potential ILS signals for any of the 10 possible tree topologies that are derivable for 4 lineages. TE analyses have already successfully demonstrated the monophyletic origin of Euarchontoglires [3] and confirmed the Primatomorpha group [4]. By applying TEs as clade markers, this study aims to clarify the internal phylogeny of Euarchontoglires, especially the still controversial position of tree shrews (Scandentia), and understand the impact of ILS that probably led to most of the published conflicting tree topologies.

## 2. Materials and Methods

**Figure 1:**
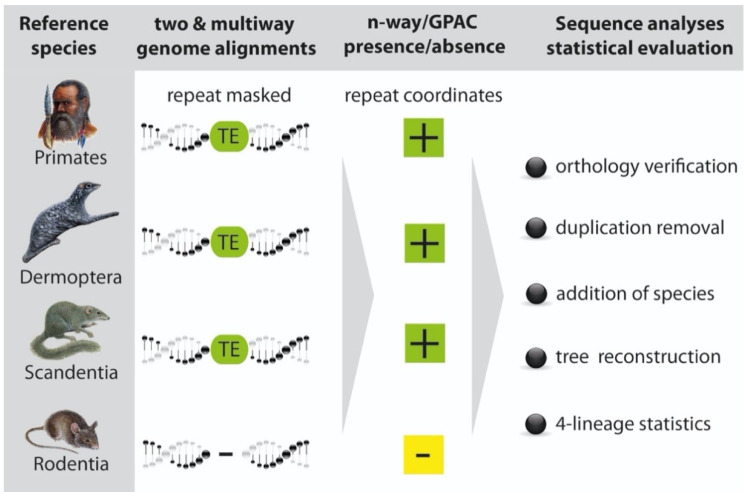
Procedure to screen for phylogenetically diagnostic TE presence/absence patterns. Reference species are shown on the left. We applied multi-way and combinations of multiple 2-way genome screenings for diagnostic TEs. All investigated genomes were previously repeat masked. Repeat coordinates were sorted in the genome presence/absence compiler (GPAC) (multi-way genome alignments) and 2-n-way (combinations of 2-way alignments) for their presence (+) or absence (−). In addition to presence/absence patterns, 2-n-way also provided the necessary sequence alignments that were carefully checked manually to verify orthology and remove duplicated loci. For GPAC, we compiled and verified alignments previously retrieved from genomic coordinates. Additional species needed for verifying the consistent presence of elements in each group were added via manual blast screening. The final steps of analysis involve tree reconstruction and 4-lineage statistics (4-LIN) to determine the significance of the trees.

In this study, a comprehensive, genome-wide screening of informative TEs for humans (*H. sapiens*), colugo (*G. variegatus*), Chinese tree shrew (*T. belangeri chinensis*), mouse (*M. musculus*), and guinea pig (*Cavia porcellus*) was carried out (Figure 1).

To determine which TE types were active during the early diversification of Euarchontoglires (only elements active in the genomes of ancestral lineages provide diagnostic phylogenetic presence/absence signals in present species), we examined the activity profiles of TEs using TinT (Transpositions in Transpositions [22]). The analysis showed that LTRs and LINE1s were the most active TEs during Euarchontoglires diversification. Element types and subfamilies screened for and analyzed in the present study are shown in Supplementary Table S1. To search for and analyze LTR and LINE1 (with ≤25 nt truncated 3′-end) presence/absence patterns, we used the repeat soft masked genomes of the Genome Browser, University of California, Santa Cruz (UCSC) (https://genome.ucsc.edu, accessed on 15 March 2022) for human (GRCh38/hg38, December 2013), colugo (G_variegatus-3.0.2/galVar1, June 2014), mouse (GRCm38/mm10, December 2011), and guinea pig (Broad/cavPor3, February 2008). The Chinese tree shrew genome (GCF_000334495.1_TupChi_1.0, January 2013) was downloaded from the National Center for Biotechnology Information (NCBI). RepeatMasker files were downloaded from UCSC and NCBI and contained the exact coordinates of all detected TEs. As an initial analysis revealed substantial underrepresentation of markers in the mouse reference genome, two species, mouse and guinea pig, were used in screening rodent genomes to achieve the best possible marker representation in rodents.

We applied two strategies to screen for TEs and correct for overlaps to increase the detected number of unique, high-quality markers: (1) A multi-way genome alignment screening with the genome presence/absence compiler (GPAC) software [23] and (2) screening of combinations of pairwise genome alignments (2-ways) in the 2-n-way software suite [24]. For this project, several multi-way genome alignments were generated in cooperation with UCSC Santa Cruz and uploaded to the GPAC tool to determine the presence/absence states of TEs: (1) human (reference)/colugo/Chinese tree shrew/mouse/guinea pig; (2) colugo (reference)/human/Chinese tree shrew/mouse/guinea pig; (3) Chinese tree shrew (reference)/human/colugo/mouse/guinea pig; (4) mouse (reference)/human/colugo/Chinese tree shrew; and (5) guinea pig (reference)/human/colugo/Chinese tree shrew. The following 2-way genome alignments were generated via the 2-way module and analyzed in n-way: (1) human/Chinese tree shrew, (2) human/guinea pig, (3) colugo/human, (4) colugo/Chinese tree shrew, (5) colugo/mouse, (6) colugo/guinea pig, (7) Chinese tree shrew/human, (8) Chinese tree shrew/colugo, (9) Chinese tree shrew/mouse, (10) Chinese tree shrew/guinea pig, (11) mouse/human, (12) mouse/colugo, (13) mouse/Chinese tree shrew, (14) guinea pig/human, and (15) guinea pig/Chinese tree shrew. Additionally, we used 2-way loads from the UCSC Genome Browser for (16) human/colugo (http://hgdownload.soe.ucsc.edu/goldenPath/hg38/vsGalVar1/, accessed on 15 March 2022); (17) human/mouse (http://hgdownload.soe.ucsc.edu/goldenPath/hg38/vsMm10/, accessed on 15 March 2022); (18) guinea pig/colugo (http://hgdownload.soe.ucsc.edu/goldenPath/cavPor3/vsGalVar1/, accessed on 15 March 2022). All 2-ways were then transferred to the n-way module for analysis.

Extracted LTR and LINE1 element coordinates for human, colugo, Chinese tree shrew, mouse, and guinea pig were loaded into GPAC and n-way to perform screenings for potentially informative markers. The n-way runs were performed with standard settings and MUSCLE-based optimization. Both GPAC and n-way generated presence/absence tables for all examined elements. In both tools, the setting “display perfect” (+) or (−) states was chosen for the selection of potential diagnostic markers. Loci for which at least two species showed a presence state of a TE insertion and at least one species showed an absence state were chosen for further manual analyses.

Using MUSCLE, we reconstructed the alignment of every locus. The qualitative analysis of the sequences and manual fine tuning of alignments for potential diagnostic presence/absence cases was carried out using the Phylogenetic Data Editor (PhyDE, Version 1.0). Where possible, orthologous sequences of a second species per order (primates, scandentians, rodents) were added to the alignments from UCSC and NCBI databases (Supplementary Table S1) to ensure there was a consistent presence/absence signal among the different lineages. In instances where mouse or guinea pig sequences were too diverged to be reliably aligned, we replaced them with sequences of other rodents (see Supplementary Table S1). Additional lagomorph sequences (pika [*Ochotona princeps*] or rabbit [*Oryctolagus cuniculus*]) were added where possible to obtain additional TE presence/absence information for the fifth Euarchontoglires lineage. Based on which species sequence was available for the corresponding locus, the cow (*Bos taurus*) or other laurasiatherian species were analyzed as outgroups (Supplementary Table S1).

**Figure 2:**
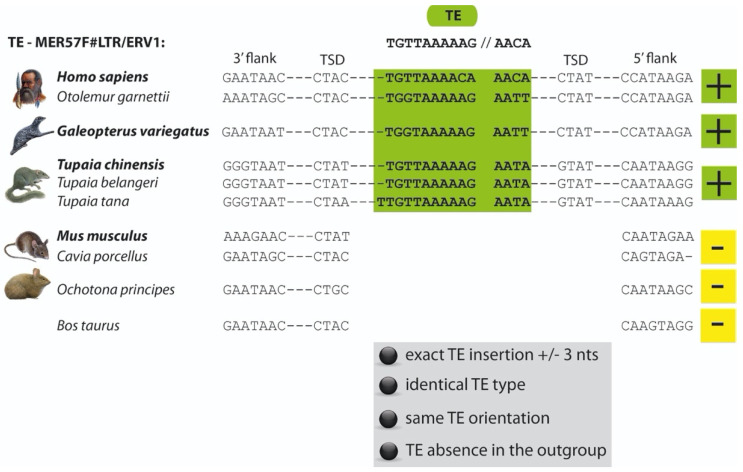
Structure of an example alignment for a MER57F#LTR/ERV1 TE (marker Euarch187). The presence of the TE is shown in green and indicated by (+). The absence state is displayed in yellow and indicated by (−). TSD indicates the TE-flanking tandem sequence duplications that appear during the insertion process of elements and are hallmarks of orthology. The stringencies of selecting a diagnostic position are given below the sequences.

For all groups examined, orthologous LTR and LINE1 insertions were assumed to be diagnostic if they fulfilled the following criteria: (1) exact presence/absence location (shifted ≤ 3 nt) among investigated species (Figure 2); (2) the same type of TE was present at a given orthologous locus; (3) the TEs at a given orthologous locus were in the same orientation; and (4) absence-state in the outgroup. All marker information was collected in an Excel table and PhyDE alignment files (Supplementary Table S1, Supplementary File S1).

Based on the presence/absence patterns of verified phylogenetically informative markers, a 1/0 matrix in NEXUS and PHYLIP format were generated, where “1” corresponds to the presence and “0” to absence (Supplementary File S2), and a lack of sequence information or a deletion in the insertion region are symbolized by “?”. Using the NEXUS 1/0 matrix, a phylogenetic network with bootstrap values of the Euarchontoglires divergence pattern was derived by neighbor-net analysis in SplitsTree [25] and a Bayesian tree reconstruction (MrBayes 3.2; ctype irreversible, mcmcngen = 20,000 samplefreq = 100 printfreq = 100 diagnfreq = 1000 [26]). The statistical significance was calculated with the 4-lineage statistics (4-LIN test; algorithm: reverse, criterion: chi-square) [27] and the incorporated KKSC test for 3-species/lineage comparisons (probabilistic test to compare reconstructed trees vs. polytomy; named after the last names of the four authors) [17]. Using the binomial distribution, a 4-LIN test estimates the probability of hybridization/introgression or ILS by evaluating the number of conflicting markers among four taxa (human, colugo, Chinese tree shrew, rodents). The PHYLIP 1/0 matrix was used to derive a Newick file with incompletely resolved “gene trees” for each character using a custom script published in [28]. ASTRAL-III [29] was applied to derive a quartet-based species-tree using Newick file as an input (ASTRAL [30] with bipartitions approach, ASTRAL_BP [28]). Bootstrap analysis was performed with 1000 pseudoreplications.

## 3. Results and Discussion

**Figure 3:**
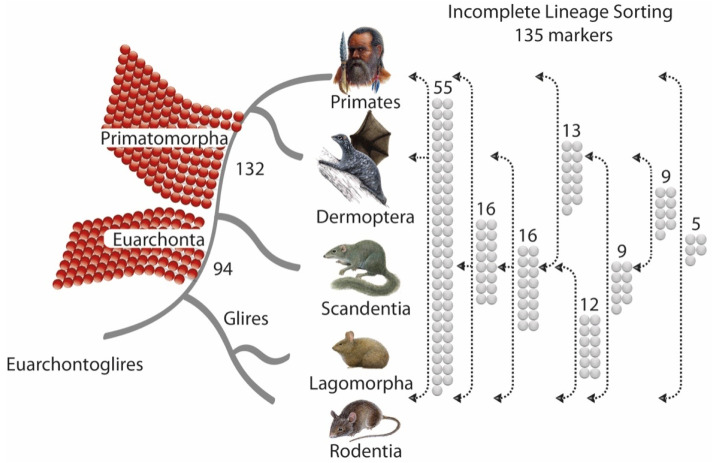
Phylogenetic reconstruction derived from TE insertion presence/absence patterns and statistically analyzed using the 4-LIN tool. Primatomorpha received the most presence/absence support (132 TE insertions), followed by Euarchonta by 94 diagnostic TEs. All markers supporting conflicting tree topologies (together 135) are indicated as grey balls with respective numbers and relationships. Branches assigned by black arrowheads show the presence of shared grey balls (orthologous insertions). All other species represent their absence. For example, nine TE insertions were present in Dermoptera and Scandentia and absent in Primates and Glires. Lagomorpha only accounted for 299 (including ten rodent-lagomorph conflicting patterns) of the 361 TE markers shown here (for details see text). The complete presence/absence matrix is given as Supplementary Table S1. We did not screen for monophyly markers of Glires and Euarchontoglires, monophyly markers of euarchontogliran orders, or phylogenetic signals within them.

For this study, we restricted our analysis to TE types that were active around the diversification of Euarchontoglires [31]. A total of 2,684,169 LTR and 338,747 LINE1 loci were initially investigated. We then extracted and manually analyzed 756 and 731 potentially informative markers, respectively, from the GPAC and 2-n-way runs. After removing duplications, our analyses revealed a total of 361 informative TE signals. Of these, 132 markers supported the Primate-Dermoptera order affiliation (Primatomorpha; see [4]), and 94 of them merged Primates, Dermoptera, and Scandentia in the clade Euarchonta (Figure 3, red phylogenetic diagnostic markers). However, the remaining 135 markers provided conflicting support for all possible alternative order affiliations (Figure 3, grey ILS markers). Within Euarchontoglires, rodents probably split off ~93.2 mya, and Scandentia diverged ~88 mya [32]. This short internode, connected to a population bottleneck, strongly suggests the possibility of ILS events [17]. Considering the virtually homoplasy-free nature of the TE presence/absence marker system [20,21], ILS is the most probable source of these controversial TE signals; however, we did not detect significant hybridization/introgression signals in the 4-LIN statistical test.

Due to the current limitation of customized statistical tests for simultaneous comparisons of only four lineages, we did not include representatives of the order Lagomorpha in our initial screening in this study. Subsequently, we added their sequences to the alignments of markers where possible. In so doing, we were then also able to identify the presence or absence state of 299 of the 361 markers in lagomorphs (Supplementary Table S1). In 289 cases, lagomorphs exhibited the same TE state as rodents, agreeing with their universally accepted shared Glires ancestry (Supplementary Table S1, [33]).

**Figure 4:**
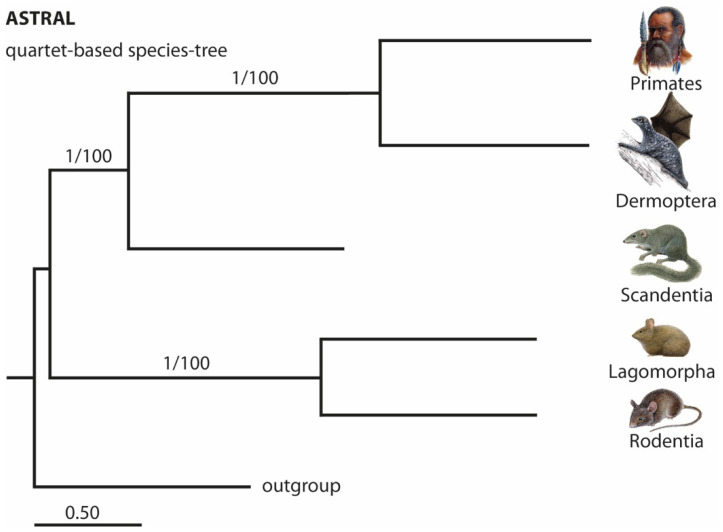
ASTRAL_BP quartet-based species tree for the 361 retrotransposon markers. Branch labels are posterior probabilities and bootstrap values, respectively. Branch lengths in coalescent units are indicated by a scale bar.

An initial evaluation of the significance of the Euarchontoglires order affiliations based on the distribution of phylogenetic signals was performed using the 4-LIN test [27]. For this, we uploaded the number of markers for each affiliation (Figure 3) to http://retrogenomics.uni-muenster.de:3838/hammlet/, accessed on 15 March 2022 (y11-y(44): 16,12,9,9,16,5,13,55,132,94, for primates, dermopterans, scandentians, and rodents, see hammlet) and found a significant support for the tree shrew as a sister group of Primatomorpha (Figure 3, 4-LIN: *p* < 1 × 10^−64^; KKSC: *p* < 6.5 × 10^−45^ for Primatomorpha and *p* < 0.0032 for Euarchonta) resolving the long-standing controversies in reconstructing Euarchontoglires phylogenetic relationships. The Bayesian analysis [26] (Supplementary Figure S1) and ASTRAL_BP [28,29,30] (Figure 4) revealed congruent results.

To better visualize the Euarchontoglires relationships, we also performed a neighbor-net analysis of TE presence/absence patterns (Supplementary Figure S1, [25]). SplitsTree has already proven to be well-suited to providing excellent visualization of phylogenetic conflicts in the form of a two-dimensional net structure (e.g., [15]). According to the prevailing view, the Primatomorpha and Glires monophyly received reliable support (100% bootstrap each) [4,33]. The proximity of the Chinese tree shrew to Primatomorpha was strongly supported, although an alternative basal position of the tree shrew within Euarchontoglires received some support.

When using TE insertion patterns to reconstruct phylogenetic relationships, it is important to design a non-biased screening strategy, and to use somewhat equal genome coverage and qualities for each possible lineage affiliation [17]. However, due to differences in genomes and variations in 2- and multi-way genome alignments that one cannot avoid, this prerequisite is only approximately achieved even in well-sequenced genomes. To minimize the risks of bias associated with the qualities of multi-way and 2-way alignments in the present study, we combined two screening strategies: multi-way screening (GPAC) and screening of 2-way combinations (n-way). We found TE markers supporting all possible order affiliations. Nevertheless, there was still a dominant and highly significant phylogenetic signal (Figure 3; red balls) that was not obscured by noise (Figure 3; grey balls) in contrast to other results derived from sequence analyses [13]. Our results, shown in an ASTRAL_BP tree reconstruction (Figure 4; 361 TE markers), generally agree with those of the previously identified tree shrew position based on protein-coding gene sequences [5,7] and ultraconserved elements [34].

## 4. Conclusions

In summary, we present highly significant evidence supporting a phylogenetic tree of Euarchontoglires that merges primates and dermopterans (132 markers) with their natural sister group of scandentians (94 markers) and places Glires at the basal position within Euarchontoglires. All alternative phylogenetic tree topologies, including those found in other published reports, were supported by far fewer, non-significant, numbers of TE insertions (in total 135 markers). The diversity of these conflicting patterns did not obscure the true phylogenetic tree of Euarchontoglires, which, according to both the 4-LIN and KKSC tests, was significantly supported by our data. To successfully use this marker system in such a phylogenetic grey zone, it was essential to collect sufficient genome-wide data from all possible directions of tree topologies by alternating reference species multiple times for separate analyses. However, due to existing sequence quality differences and genome alignment uncertainties due to different levels of sequence divergences, re-analyses might vary slightly but will certainly lead to the same conclusions.

## Data Availability

All data are present in the Supplemental Files.

## Supplementary Materials

The following supporting information can be downloaded at: https://www.mdpi.com/article/10.3390/genes13050774/s1, Figure S1: Neighbor-net and MrBayes analyses; Table S1: Presence/absence table; File S1: Alignments; File S2: 1/0 matrix.
