# Supplementary figures and images for: Euarchontoglires Challenged by Incomplete Lineage Sorting
Source: Genes (Basel). 2022 Apr 27;13(5):774. doi: 10.3390/genes13050774 (PMC9141288; doi:10.3390/genes13050774)

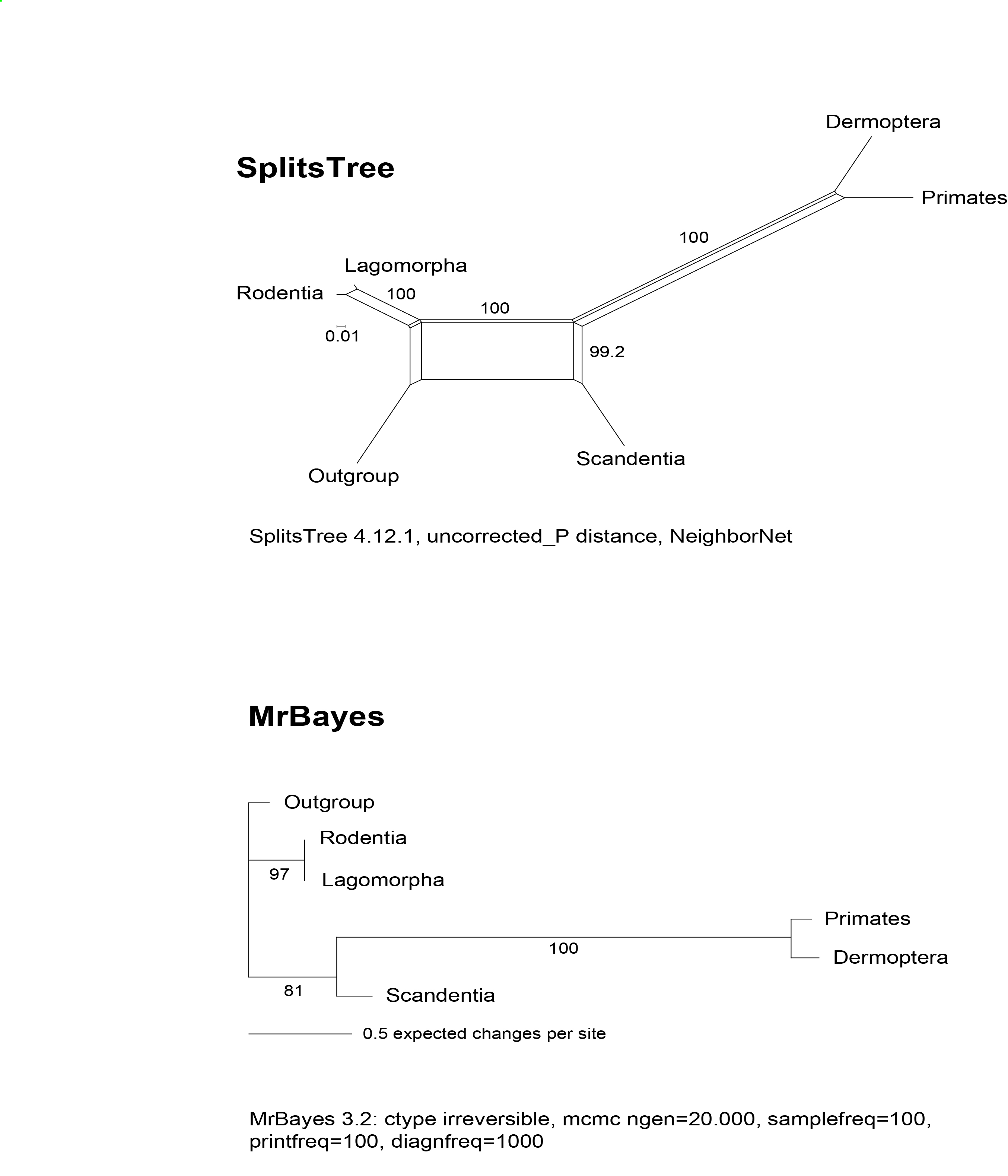

Supplement: Supplementary file 1 [file genes-13-00774-s001.zip › Doronina_Supplementary_Files/Supplementary_Figure_S1.jpg]
